# Acute Generalized Exanthematous Pustulosis following SARS-CoV-2 Virus: Remdesivir as a Suspected Culprit

**DOI:** 10.1155/2022/9880827

**Published:** 2022-08-10

**Authors:** Fatemeh Mohaghegh, Parvaneh Hatami, Zeinab Aryanian, Farahnaz Fatemi, Zeinab Mohseni Afshar

**Affiliations:** ^1^Department of Dermatology, Skin Diseases and Leishmaniasis Research Center, Isfahan University of Medical Sciences, Isfahan, Iran; ^2^Autoimmune Bullous Diseases Research Center, Tehran University of Medical Sciences, Tehran, Iran; ^3^Department of Dermatology, Babol University of Medical Sciences, Babol, Iran; ^4^Clinical Research Development Center, Imam Reza Hospital, Kermanshah University of Medical Sciences, Kermanshah, Iran

## Abstract

Acute generalized exanthematous pustulosis (AGEP) is an exanthematous condition, predominantly occurring as a result of drug reactions. We, hereby, present the first case of AGEP following treatment with remdesivir in a patient with COVID-19, without hydroxychloroquine use, which serves as a reminder to consider remdesivir as a possible causative agent when dealing with AGEP presentation in COVID patients.

## 1. Introduction

Coronavirus disease 2019 (COVID-19), caused by the severe acute respiratory syndrome coronavirus 2 (SARS-CoV-2) has been well-known for its multisystemic involvement; besides respiratory manifestations, mucocutaneous symptoms have been among the most common presentations of SARS-CoV-2 infection [1]. Cutaneous manifestations of SARS-CoV-2 infection can range from erythematous or maculopapular eruptions and urticaria to blisters, petechiae, and livedo reticularis [2]. Skin involvement can occur as the sole manifestation of COVID-19 infection, intervene during the course of infection, or appear after the infection has subsided [3, 4]. On the other hand, cutaneous reactions can be a manifestation of a new-onset dermatosis or an exacerbation of an existing condition [5]. The underlying cause of this phenomenon can be the virus's direct invasion into the skin and mucosal surfaces, the immunologic inflammatory response elicited by the virus, or the side effects of therapeutics used in the settings of SARS-CoV-2 infection [6, 7].

Acute generalized exanthematous pustulosis (AGEP) is an exanthematous condition with an abrupt onset, which predominantly occurs as a result of a drug reaction. In fact, other factors such as infections, vaccinations, chemicals contact, and insect bites can also be triggering factors for AGEP [5]. Since the beginning of the COVID-19 pandemic, an increased rate of AGEP has been reported, which could be attributed to the high prevalence of the SARS-CoV-2 virus as the causative pathogen, or the result of medications used in these settings. Here, we report a case of COVID-19 associated with AGEP with significant diagnostic challenges and complications, and we present a brief literature review.

## 2. Case Presentation

**Figure 1:**
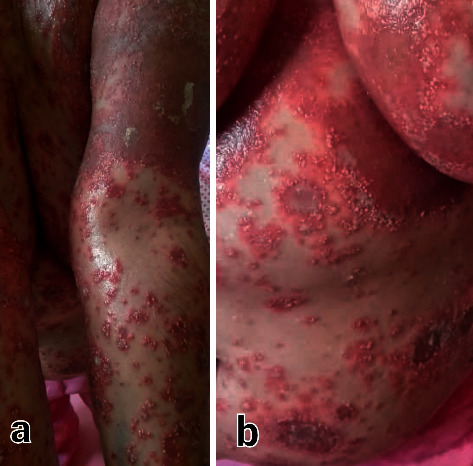
Generalized pustular eruptions with an erythematous base on the body (b) and extremities (a).

A 62-year-old woman presented to the dermatology clinic with a generalized pruritic skin rash. She mentioned a history of SAR-CoV-2 infection one month earlier, for which she had undergone remdesivir treatment. Two days after the completion of treatment, she developed cutaneous reactions beginning in the trunk and extending to the extremities. She also mentioned a history of Addison's disease, for which she was taking prednisolone and fludrocortisone. On physical examination, widespread pustular eruptions with an erythematous base were covering all of her body surfaces except for the head and the neck area (Figure 1). The hair and nails were intact. No mucosal involvement was detected. She was started on high-dose oral prednisolone and cyclosporine in conjunction with acitretin and topical emollient. A few days later, she returned to the clinic with fever and toxicity. Laboratory evaluation revealed thrombocytopenia and transaminitis. Therefore, we admitted her for further workup. We initially stopped all the drugs she was taking, including cyclosporine and acitretin, and took a skin biopsy.

**Figure 2:**
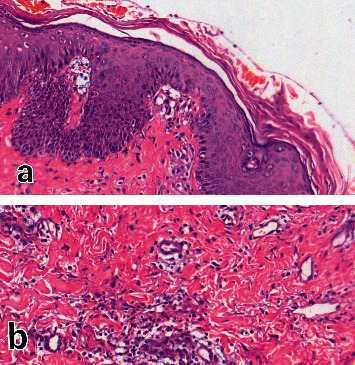
Neutrophilic parakeratosis with mild acanthosis and focal spongiosis, along with the scattered necrotic keratinocytes, ectatic capillaries, and perivascular interstitial lymphocytic and eosinophilic infiltration: H&E ×40 (a); H&E ×100 (b).

**Figure 3:**
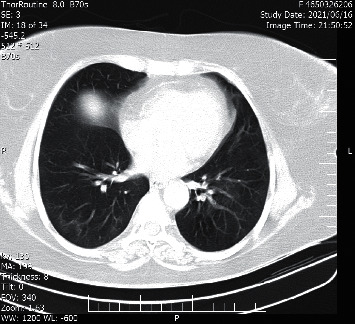
Diffuse ground-glass opacities in both the lungs compatible with the convalescent pulmonary phase of SARS-CoV-2 infection.

The histopathology report was indicative of linear neutrophilic parakeratosis with mild acanthosis and focal spongiosis, along with scattered necrotic keratinocyte, ectatic capillaries, and perivascular interstitial lymphocytic and eosinophilic infiltration in the dermis, all compatible with the diagnosis of AGEP (Figure 2). Then, we started her on methylprednisolone pulse and IVIG due to the possibility of idiopathic thrombocytopenia (ITP) in this patient. She was tested negative for COVID-19 and HIV-Ab. The chest CT scan, as was expected, showed diffuse ground-glass opacities in both the lungs compatible with a convalescent pulmonary phase of SARS-CoV-2 infection (Figure 3). Three days after the initiation of intensive therapy, platelet counts increased and liver enzymes decreased. The eruptions rapidly resolved within a few days; therefore, we switched the therapy to intravenous hydrocortisone and then to prednisolone with gradual tapering. At the time of discharge, postpustular desquamation was demonstrated. At follow-up visits, the patient did not describe any relapses.

## 3. Discussion

Acute generalized exanthematous pustulosis (AGEP) is an acute pustular dermatosis with the abrupt onset of a great number of pustules with edematous and erythematous bases [8].

Several conditions, including infections, vaccination, and medications, have been mentioned as precipitating factors of AGEP [9]. The COVID-19 pandemic has unveiled a wide range of dermatologic disorders, new-onset or flare, in SARS-CoV-2 infected patients. Therefore, this virus should also be listed among the infectious causes of AGEP [10].

Medications most commonly associated with AGEP include pristinamycin, aminopenicillins, fluoroquinolones, antimalarials, sulfonamides, terbinafine, azoles, protease inhibitors, dapsone, pantoprazole, diltiazem, corticosteroids, azithromycin, NSAIDs, and antiepileptic agents [8]. Among the common therapeutics used during a SARS-CoV-2 infection with the probability of inducing AGEP, we can name hydroxychloroquine, which is the most notorious medication for inducing AGEP, favipiravir, azithromycin, NSAIDs, protease inhibitors such as lopinavir-ritonavir, anticoagulants, and glucocorticoids [11–19]. Our patient had received azithromycin, dexamethasone, naproxen, and remdesivir for SARS-CoV-2 infection. Therefore, the onset of AGEP could be attributed to any of the mentioned agents, although there has been no definite report of remdesivir-associated AGEP.

In AGEP, the duration of drug exposure before the onset of the symptoms varied from a few hours to a few weeks depending on the causative drug [17]. Our case presented with AGEP 4 weeks after receiving COVID treatment, which was a longer latency period compared to AGEP reported in COVID-19 infected patients in the recent literature, which makes it difficult to attribute the AGEP to these medications. However, one can infer that the combination of each of these medications, including remdesivir and genetic disposition with COVID-19 induced cytokine storm, led to the delayed development of AGEP in this case. In fact, AGEP is a hypersensitivity reaction mediated by CD8+/CD4 +T cells. Keratinocyte apoptosis occurs secondary to the migration of drug-specific T cells to the skin via granzyme B and the Fas ligand mechanism [20].

Moreover, interferon-gamma and granulocyte/macrophage colony-stimulating factors released by the drug-specific T cells could lead to prolongation of the process [21].

Though AGEP might be considered as an unreported side effect of remdesivir, which has been widely used during the COVID era, it could be merely the result of the resemblance of inflammatory cytokine cascade alterations in the setting of both COVID-19 and AGEP, which let us consider these lesions as some late-onset cutaneous manifestations of COVID-19 [22]. Further reports will shed more light on this issue.

Eliminating the causative trigger, such as ceasing the drug or treating the infection, is the cornerstone of AGEP management. Other potentially useful options include moist dressings and topical antiseptics, systemic antibiotics if superinfection intervenes, and topical or systemic corticosteroids [23, 24]. However, in severe or recalcitrant cases, cyclosporine and intravenous immunoglobulin may be beneficial [25].

## 4. Conclusion

We reported a case of AGEP, which might have been triggered by COVID-19 infection itself or be considered as an unreported possible side effect of remdesivir, to emphasize the necessity of paying more attention to history-taking and clinical suspicion as key factors to reaching the correct diagnosis [26–31]. Furthermore, one can infer from our report that when dealing with skin findings, remdesivir should be kept in mind as a causative agent.

## Data Availability

The data used to support the findings of this study are available from the corresponding author upon request.

## Additional Points

*Key Clinical Message.* The authors presented the first case of AGEP following treatment with remdesivir in a patient with COVID-19 without hydroxychloroquine use which could be an important issue when dealing with cutaneous rashes, considering the widespread use of remdesivir in the COVID era.
